# Heel riser height and slope gradient influence the kinematics and kinetics of ski mountaineering—A laboratory study

**DOI:** 10.3389/fspor.2022.886025

**Published:** 2022-08-18

**Authors:** Michael Lasshofer, John Seifert, Anna-Maria Wörndle, Thomas Stöggl

**Affiliations:** ^1^Department of Sport and Exercise Science, University of Salzburg, Hallein, Austria; ^2^Department of Health & Human Development, Montana State University, Bozeman, MT, United States; ^3^Red Bull Athlete Performance Center, Salzburg, Austria

**Keywords:** human-equipment interaction, sports equipment, treadmill ergometry, winter sports, ski mountaineering, mechanical efficiency

## Abstract

In ski mountaineering, equipment and its interaction with the exercising human plays an important role. The binding, as the crucial connection between boot and ski, must ensure safe fixation during downhill skiing and a free moving heel when walking uphill. Uphill, the binding offers the possibility to adopt the height of the heel (riser height) to personal preferences and the steepness of the ascent. This possible adjustment and its influence on various biomechanical parameters are the focus of this work. For this study, 19 male leisure ski mountaineers were tested on a treadmill, ascending at a fixed submaximal speed (3.9 ± 0.4 km·h^−1^) at 8, 16, and 24% gradient and with three heel riser heights, low (0 cm), medium (3.0 cm) and high (5.3 cm). The applied biomechanical measurement systems included a 3D motion capture system in sagittal plane, pressure insoles, a with strain gauges instrumented pole, spirometry and a comfort scale. Step length and step frequency were influenced by the riser height and the gradient (*p* ≤ 0.001). The high riser height decreased the step length by 5% compared to the low riser height over all tested gradients, while steps were 9.2% longer at the 24% gradient compared to the 8% gradient over all three riser heights. The high riser height revealed a force impulse of the pole 13% lower than using the low riser height (*p* < 0.001). Additionally, the high riser height reduced the range of motion of the knee joint and the ankle joint compared to the low riser height (*p* < 0.001). Therefore, advantageous settings can be derived, with the low riser height creating proper range of motion for ankle, knee and hip joint and higher propulsion via the pole at 8%, while higher riser heights like the medium setting do so at steeper gradients. These findings are in line with the conducted comfort scale. We would not recommend the highest riser height for the analyzed gradients in this study, but it might be an appropriate choice for higher gradients.

## Introduction

Most sports are highly reliant on the equipment used in performing them. Applying the proper equipment can promote the progression of performance (Haake, 2009) and minimize the risk of injuries (Stefanyshyn and Wannop, 2015). These factors are directed by sport-specific rulebooks to ensure safe and attractive competition circumstances (Cooper and De Luigi, 2014; Müller et al., 2016; Crouch et al., 2017). However, human–equipment interaction is not trivial and can be affected by various parameters (Stefanyshyn and Wannop, 2015).

To serve changing demands, some sports offer the possibility to adapt the equipment, even during exercise. One of these sports is ski mountaineering (skimo). To serve the demands of walking uphill and skiing downhill, the boots, binding and skis have specific features. For example, the heel binding enables the user to alter a heel riser height according to personal preferences in the given environment when walking uphill. General recommendations indicate using a higher riser height at steeper slope gradients to keep the foot in a more horizontal position when walking uphill to sustain an upright posture and reduce the stretch to the calves (Vives, 1999; Winter, 2001). A common exception are race bindings which do not provide possibilities for changes in riser height. These bindings offer one fixed height, which is comparable to a medium riser height in touring or recreational bindings (House et al., 2019) and is used universally in flat, but also steep terrain.

Nevertheless, the justification to various recommendations for skimo riser height is more reliant on experts' opinions and general experience than on empirical evidence. Scientifically, skimo is a rather young sport with only a few topic areas thoroughly researched. Skimo racing has been shown to be one of the most strenuous endurance exercises at the elite level (Duc et al., 2011; Praz et al., 2014; Fornasiero et al., 2018; Gaston et al., 2019; Lasshofer et al., 2021). Part of the performance improvement aspect is the enormous development in training and equipment over the last decades (Bortolan et al., 2021), halving the metabolic cost of moving with skis on snow (Formenti et al., 2005). To reach a summit, steeper slope angles provide lower vertical energy cost and are therefore mechanically more efficient, compared to flatter slope angles. While walking speed does not influence the vertical energy cost in slightly inclined terrain, maintaining higher speed in steep terrain is associated with lower vertical energy cost (Praz et al., 2016a,b). Movement patterns are highly influenced by terrain, therefore the foot sole loading pattern clearly distinguishes between a direct ascent and traversing (Haselbacher et al., 2014). The combined effects of equipment and equipment variations in skimo have not been studied in depth. Tosi et al. (2009) observed only a small influence of adding weight to the ankle concerning energy cost. Adding one percent of body weight to the ankle during skiing only resulted in an increase of energy cost by 1.7%.

The present study was designed to extend the knowledge on the biomechanics of skimo, verify the experience-based opinions and provide further understanding of human–equipment interaction in this sport.

Therefore, the goal of this study was to compare the influence of different heel riser heights and gradients on kinematic, kinetic variables, mechanical efficiency and comfort during treadmill skimo. We hypothesize that higher riser heights are beneficial on steeper slope gradients (e.g., longer step cycle, more horizontal foot position, more upright body position, pole application) and low riser heights on low slope gradients.

## Methods

### Participants and protocol

As inclusion criteria, participants were male, between 18 and 50 years old, practice skimo regularly, do not participate in skimo races and apply different available riser heights regularly while walking uphill. Nineteen participants were included in the study, whose anthropometric data, equipment characteristics and training habits are shown in Table 1. All participants took part voluntarily and signed a letter of agreement. The study was approved by the ethical committee of the University of Salzburg (EK-GZ: 36/2018).

**Table 1:** Age, anthropometrics, equipment and training.

	**Overall (*****n*** = **19)**		
	**Mean ±SD**	**Min**	**Max**
Age [yr]	34.0 ± 7.3	21	49
Body height [cm]	179.5 ± 8.7	159	195
Body mass [kg]	78.0 ± 8.3	58.5	94.8
BMI [kg·(m^2^)^−1^]	24.3 ± 2.9	18.1	30.9
VO_2peak_ [ml·min^−1^·kg^−1^]	57.1 ± 5.8	48.1	65.8
Ski length [cm]	172 ± 8	160	188
Ski length in % of body height [%]	96 ± 5.4	87.7	103.9
Ski width [mm]	84 ± 13	60	106
Pole length [cm]	129 ± 6	120	145
Pole length in % of body height [%]	72.2 ± 2.7	68.4	78.6
Total training volume [h/week]	8.3 ± 4.3	2	20
Skitours [n/month]	9.4 ± 5.6	2	20
Elevation gain [m/tour]	1,076 ± 224	750	1,500

*SD, standard deviation; BMI, body mass index*.

**Figure 1:**
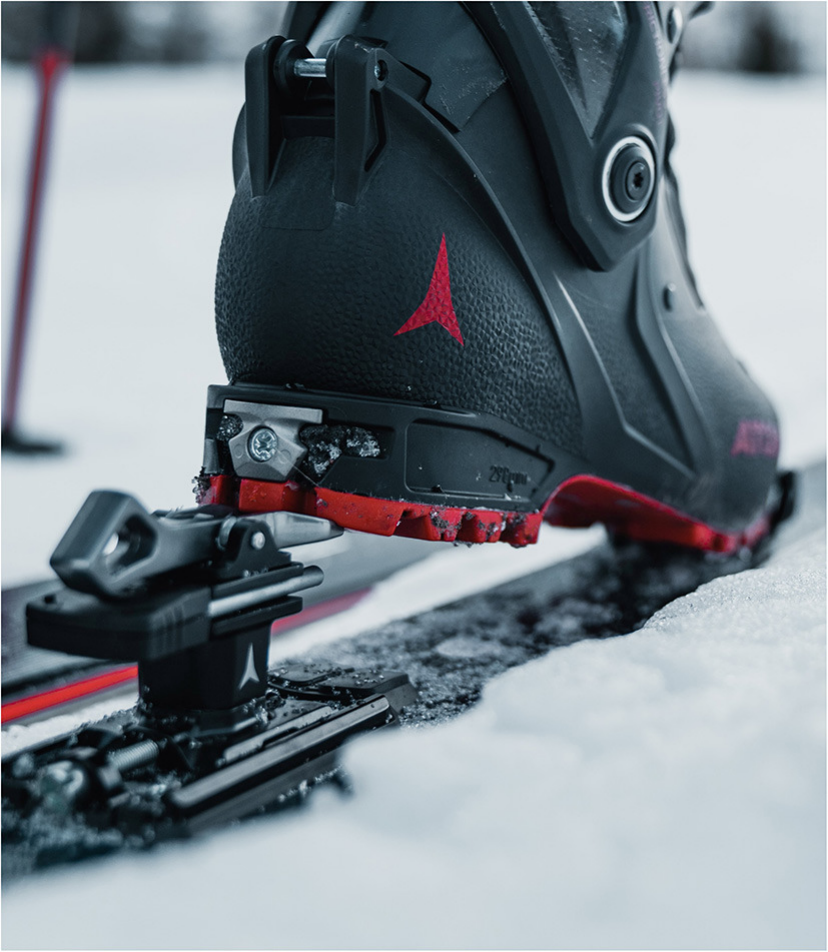
Showing the used skimo binding with the medium riser height applied. By flipping the support area the low or high riser height could be applied. © 2020 | Atomic Austria GmbH.

Participants walked on a h/p/cosmos Saturn treadmill (h/p/cosmos sports medical GmbH, Germany, size 300 × 125 cm) for two sessions using standardized skimo equipment. An Atomic Backland Tour binding was mounted on a 170 cm long Atomic Backland 78 ski (Atomic Austria GmbH, Austria). Atomic Backland Sport boots (Atomic Austria GmbH, Austria) in various sizes with a reported range of motion of 74° were used for all tests (Atomic, 2021). Short standard skimo climbing skins (0.3 m) were mounted below the area of the binding to ensure sufficient grip but not to fully restrict gliding, which then provided a perception similar to walking on snow. Instrumented poles, in which the lengths were individually adjusted to fit the participants' habitual pole length, were used for the two measurement sessions. The first test was a skimo specific performance test to determine physiological fitness, determine the walking speed for the second session and to become familiar with the movement characteristics on the treadmill. This test was a combination of an incremental test at a 16% gradient (0.4 km·h^−1^ increment every 4 min with a 30 s break to take the lactate sample starting at 2.6 km·h^−1^ until reaching ≥4 mmol·L^−1^ blood lactate) and after a passive 3-min break, a ramp test at 24% gradient (0.4 km·h^−1^ increment every minute starting at 2.6 km·h^−1^ until reaching exhaustion). The second session was a duration test, which had to be at minimum 72 h and at maximum 2 weeks after the first session. The walking speed, which was on average 4.0 ± 0.5 km·h^−1^, was derived from the first session and corresponds to the speed at 1.5 mmol·L^−1^ blood lactate. The testing included three times 15 min at 8, 16, and 24% gradient in this order at the given consistent speed, while each block was split in three times 5 min intervals at the three available riser heights being applied randomly. The break between the 5 min intervals was 1 min to change the riser height, and between different gradients a break of 2 min was necessary to change the gradient and the riser height. The characteristics of the riser height were low (0.0 cm), medium (3.0 cm), and high (5.3 cm) (Figure 1). Only the last minute of each 5 min interval was recorded and the middle 20 strides out of this minute were further analyzed.

### Measuring setup

Participants were equipped with three different biomechanical systems. The Moticon sensor insoles (Moticon ReGo AG, Germany) recording at 100 Hz with an integrated 3+3-axis IMU were used to measure foot pressure distribution perpendicular to the insole, apparent as vertical ground reaction force. Gait and foot sole loading patterns were computed with the Moticon Science software. The software creates an automated report over selected steps including step frequency, step length along the surface, timing parameters (cycle, step, single stance, double stance and swing time) force and pressure parameters. To do so, the input parameters are total force, pressure distribution and acceleration along the x-axis. Maximum ground reaction force data were intercepted in F_peak_ and F_max_, where F_peak_ was the peak value at initial ground contact and F_max_ the overall maximum value typically occurring during push off. The force impulse was calculated by multiplying contact time with the mean force during ground contact.

An in-house built instrumented pole (University of Salzburg, Austria) with a strain gauge force transducer (ME-Meßsysteme GmbH, Hennigsdorf, Germany) was used for measuring pole forces directed along the pole. Sampling frequency for this system was 200 Hz and data was transmitted wirelessly via Bluetooth to a Smartphone application (Sentax, Sweden). A 12 Hz second order low pass Butterworth filter was applied to the raw data before further data processing and obtaining pole ground contact time, mean and maximum ground reaction force.

Kinematic data was captured by a Qualisys Miqus 3D motion capture system recording at 100 Hz (Qualisys AB, Sweden). The system was applied to assess data in sagittal plane. Markers were placed on the boot (toe, heel and ankle), knee (lateral joint line), hip (greater trochanter), and shoulder (acromion) of the participants. Boot markers represented the foot, but did not necessarily correspond with anatomical structures or provide the same possibility to move because of the rigidity of the boot shell. The ankle marker was placed at the pivot point of the boot and the criterion for the heel and toe marker were to be at the same height measured from the ground. Additionally, two markers were placed on the pole tube, 20 cm and 35 cm above the pole tip. Joint angles were defined based on the following marker positions: Ankle angle: angle between toe marker, ankle marker and knee marker; Knee angle: angle between ankle marker, knee marker and greater trochanter marker; Hip angle: angle between knee marker, hip marker and shoulder marker. The ROM (range of motion) was calculated as the difference between maximum and minimum joint angle during each analyzed cycle. The pole angle was measured as the angle between the pole and the treadmill at initial contact of the pole. The torso angle, defined as the angle in sagittal plane between a horizontal line and the line between the shoulder marker and the hip marker, and the distance between hip and toe were captured during initial contact of the foot. The distance between hip and toe assesses the hip's horizontal distance relative to the toe, which corresponded to the pivot point of the binding. The foot angle, defined as the angle in sagittal plane between the horizontal axis and the line between the toe and heel marker, described the foot position during ground contact relative to horizontal. When applying the lowest riser height setting, respectively, no riser height, the line between the two relevant markers was parallel to the surface. Negative values indicate that the heel marker was on a lower level than the toe marker in the global space, while positive values indicate that the heel marker was higher than the toe marker. Since the 3D motion capture system was applied unilaterally, foot forces and pole forces were also analyzed unilaterally. Assuming skimo being a cyclical movement, minor right-left asymmetries were expected to be neutralized over the tested cohort and therefore no loss of data quality was presumed.

A portable metabolic system, a Cosmed K5 (Cosmed, Rome Italy) was applied in breath-by-breath mode to measure oxygen uptake. Metabolic rate was taken from the Cosmed data output as well. The system was calibrated before each test following the instruction manual, the facemasks were fitted properly and a fan in combination with opened windows ensured fresh air circulation. Skimo specific maximum oxygen consumption was defined as a mean value over 15 consecutive breaths at the end of the ramp protocol with a flattening VO_2_ slope and respiratory exchange ratio being > 1.05 or rating of perceived exertion (Borg 6–20) being > 18. Lactate samples were taken by qualified researchers from an ear lobe before, during and after the test and the 20 μl blood sample was analyzed by an EKF-Diagnostics Biosen C-line system (EKF-diagnostic GmbH, Germany).

Mechanical efficiency was calculated similar to Praz et al. (2014, 2016a,b):


$$\begin{array}{l} Mechanical\;efficiency=vertical\;mechanical\;power/metabolic\;rate \end{array}$$

Where vertical mechanical power followed the equation:


$$\begin{array}{l} Vertical\;mechanical\;power=m^{*}g^{*}sin{\left(arctan\left(\theta \right)\right)}^{*}v \end{array}$$

with m being the mass of the athlete + the equipment, g the acceleration of gravity, θ the gradient given in % and v the walking velocity (m·s^−1^).

Additionally, after each condition participants were asked how comfortable the applied riser height was. For this purpose, a comfort scale (1–10) was used, with 1 representing a very uncomfortable situation and 10 representing a very comfortable situation.

### Statistics

For statistical calculations, SPSS Version 27 (IBM Cooperation, USA) was used. A multifactorial ANOVA with repeated measurements was applied for determination of main effects of gradient and riser height, while a one-way ANOVA was used for detailed analysis within the separate gradients. For pairwise comparisons, a Bonferroni correction was applied. Whenever sphericity was not given (Mauchly Test *p* < 0.05), Greenhouse-Geisser correction was applied for within-subjects effects. Alpha value for significance was defined as <0.05. Partial Eta squared (${\text{eta}}_{\text{p}}^{2}$) is reported as effect size.

## Results

Only complete datasets were analyzed for each dependent variable, which resulted in a sample size of 18 for the kinematic parameters and foot pressure and 16 for parameters related to the instrumented pole. The loss of data in one case was due to fatigue and incomprehensible technical issues leading to lost data in the other cases.

Table 2 summarizes kinematic and kinetic parameters, mechanical efficiency and comfort scale for each situation (three riser heights for each of the three slope gradients) including the main effects and interaction effects.

**Table 2:** Kinematic and kinetic data.

					**ANOVA (** ***p*** **-value/** ${\text{eta}}_{\text{p}}^{2}$ **)**		
		**low RH**	**med RH**	**high RH**	**GR**	**RH**	GR*RH
Distance trochanter—toe [m]	8%	0.51 ± 0.05	0.48 ± 0.05	0.45 ± 0.07			
	16%	0.56 ± 0.05	0.54 ± 0.06	0.52 ± 0.06	**<0.001/0.94**	**<0.001/0.75**	**0.003/0.23**
	24%	0.59 ± 0.05	0.57 ± 0.06	0.55 ± 0.06			
Torso angle [°]	8%	86 ± 5	87 ± 5	87 ± 4			
	16%	82 ± 5	83 ± 5	84 ± 5	**<0.001/0.83**	**<0.001/0.79**	**<0.001/0.32**
	24%	76 ± 7	78 ± 7	79 ± 6			
Pole angle [°]	8%	68 ± 5	69 ± 7	69 ± 6			
	16%	66 ± 6	66 ± 7	66 ± 7	**<0.001/0.71**	0.07/0.14	**0.036/0.14**
	24%	61 ± 6	62 ± 7	63 ± 7			
Pole contact time [s]	8%	0.68 ± 0.09	0.68 ± 0.09	0.67 ± 0.08			
	16%	0.75 ± 0.1	0.74 ± 0.1	0.7 ± 0.1	**<0.001/0.86**	**<0.001/0.68**	**0.005/0.23**
	24%	0.82 ± 0.09	0.77 ± 0.09	0.77 ± 0.1			
Pole Fmax [N]	8%	43 ± 12	42 ± 11	44 ± 11			
	16%	55 ± 15	52 ± 14	52 ± 13	**<0.001/0.85**	**0.005/0.31**	0.072/0.14
	24%	71 ± 17	66 ± 15	65 ± 15			
Sole Fmax [N]	8%	936 ± 126	898 ± 126	886 ± 131			
	16%	968 ± 142	939 ± 134	913 ± 148	**0.001/0.43**	**<0.001/0.48**	0.606/0.04
	24%	984 ± 112	973 ± 135	951 ± 138			
Sole Fpeak [N]	8%	595 ± 108	642 ± 129	680 ± 140			
	16%	583 ± 116	622 ± 95	645 ± 104	0.292/0.07	**<0.001/0.64**	0.309/0.07
	24%	562 ± 96	648 ± 137	641 ± 108			
Cycle time [s]	8%	1.32 ± 0.12	1.28 ± 0.11	1.26 ± 0.13			
	16%	1.37 ± 0.12	1.34 ± 0.13	1.31 ± 0.13	**<0.001/0.48**	**<0.001/0.68**	0.547/0.04
	24%	1.38 ± 0.13	1.35 ± 0.12	1.34 ± 0.13			
Step time [s]	8%	0.79 ± 0.08	0.78 ± 0.07	0.77 ± 0.09			
	16%	0.82 ± 0.07	0.8 ± 0.07	0.78 ± 0.08	**0.016/0.26**	**<0.001/0.37**	0.579/0.04
	24%	0.83 ± 0.07	0.81 ± 0.07	0.8 ± 0.08			
Single stance time [s]	8%	0.66 ± 0.06	0.64 ± 0.06	0.63 ± 0.08			
	16%	0.68 ± 0.06	0.67 ± 0.07	0.65 ± 0.07	**0.002/0.36**	**<0.001/0.61**	0.651/0.03
	24%	0.69 ± 0.06	0.67 ± 0.06	0.66 ± 0.06			
Double stance time [s]	8%	0.13 ± 0.02	0.14 ± 0.02	0.14 ± 0.03			
	16%	0.14 ± 0.03	0.13 ± 0.02	0.13 ± 0.03	0.289/0.07	0.947/ <0.01	0.327/0.07
	24%	0.14 ± 0.03	0.14 ± 0.03	0.14 ± 0.03			
Swing time [s]	8%	0.53 ± 0.05	0.5 ± 0.06	0.48 ± 0.06			
	16%	0.55 ± 0.06	0.54 ± 0.06	0.53 ± 0.06	**<0.001/0.53**	**<0.001/0.63**	0.09/0.11
	24%	0.55 ± 0.06	0.54 ± 0.06	0.53 ± 0.06			
Foot angle [°]	8%	−4.5 ± 0.5	4.1 ± 0.6	8.4 ± 0.8			
	16%	−9.2 ± 0.5	−0.6 ± 0.6	3.7 ± 0.8	**<0.001/1**	**<0.001/0.99**	0.153/0.1
	24%	−13.5 ± 0.5	−5 ± 0.6	−0.7 ± 0.8			
Mechanical efficiency	8%	0.1 ± 0.01	0.09 ± 0.01	0.09 ± 0.01			
	16%	0.14 ± 0.01	0.14 ± 0.01	0.14 ± 0.02	**<0.001/0.98**	0.756/0.02	0.186/0.09
	24%	0.17 ± 0.02	0.17 ± 0.02	0.17 ± 0.02			
Comfort (1–10)	8%	8.6 ± 1.3	7.0 ± 1.7	4.8 ± 2.6			
	16%	6.6 ± 2.0	7.5 ± 1.5	6.6 ± 1.8	**0.016/0.24**	**<0.001/0.41**	**<0.001/0.41**
	24%	5.2 ± 1.9	6.5 ± 2.2	6.1 ± 2.1			

*Mean ± standard deviation; RH, riser height; GR, gradient; GR × RH = interaction effect between gradient and riser height. Significant results are presented in bold*.

### Cycle characteristics

**Figure 2:**
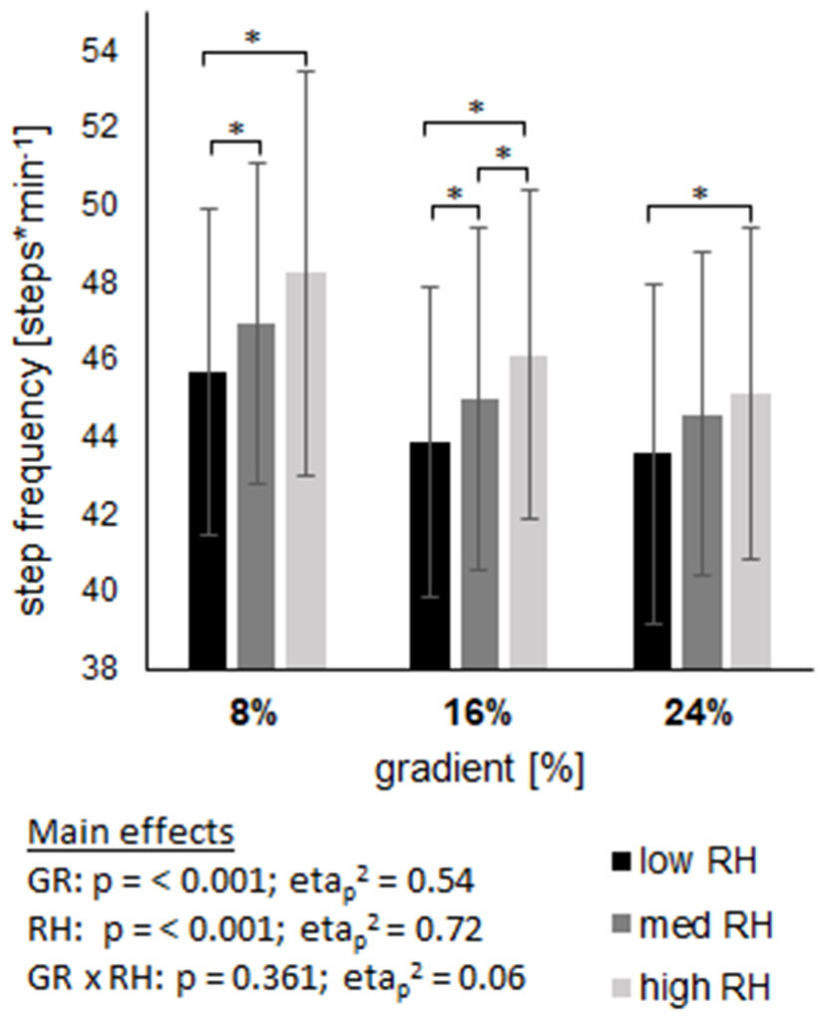
Shows the step frequency across three gradients (GR), each including three riser heights (RH). Data are displayed as mean value ± standard deviation. GR × RH represents their interaction effect. The asterix (*) labels pairwise comparisons within each grade, with the level of significance (p) set at <0.05.

**Figure 3:**
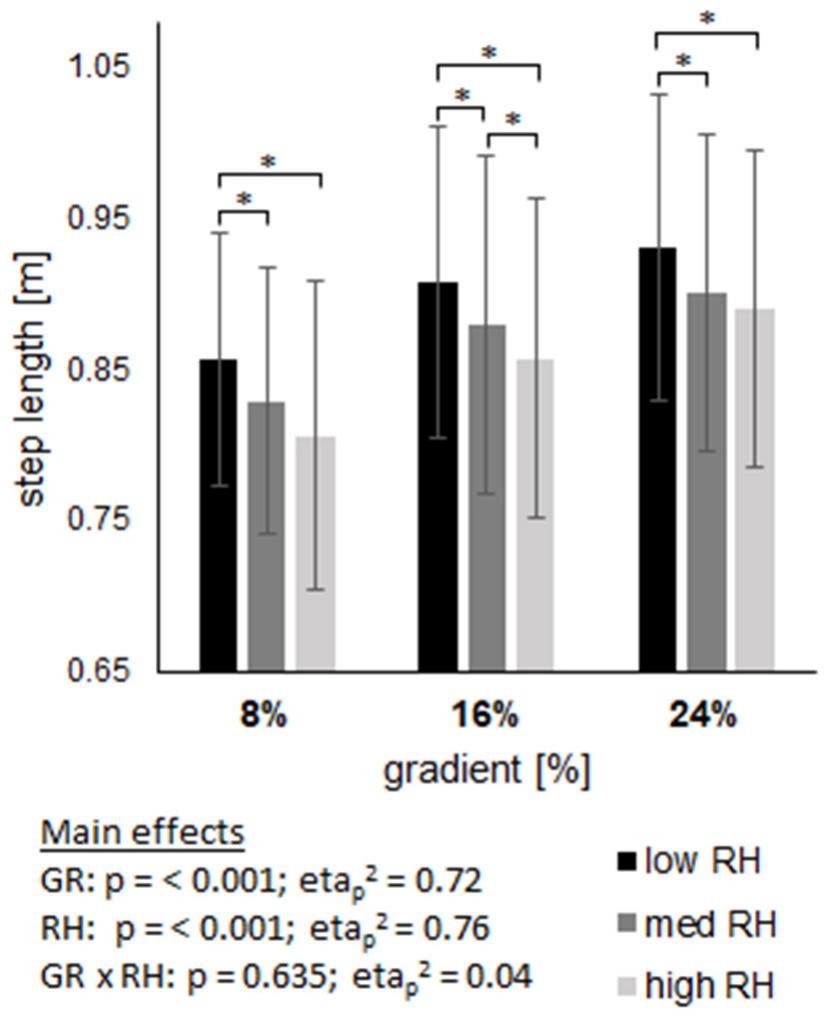
Shows the step length across three gradients (GR), each including three riser heights (RH). Data are displayed as mean value ± standard deviation. GR × RH represents their interaction effect. The asterix (*) labels pairwise comparisons within each grade, with the level of significance (p) set at <0.05.

Both, step length and step frequency revealed a main effect of gradient and riser height (both, p <0.001) without an interaction effect (Figures 2, 3). On average over all gradients, step length decreased by 5% from low to high riser height. On average over all riser heights, step length increased by 9.2% from 8% gradient to 24% gradient. In contrast to step length, step frequency was increased by 4.8% from low to high riser height and was reduced by 5.4% from 8% gradient to 24% gradient.

The overall reduction of cycle time by 4% from low to high riser height over all gradients (*p* < 0.001) is a result of the reduction of step duration (−3.7%; *p* < 0.001) and swing duration (−5.5%; *p* = <0.001). No interaction effects were found for cycle timing characteristics.

### Leg and pole characteristics

**Figure 4:**
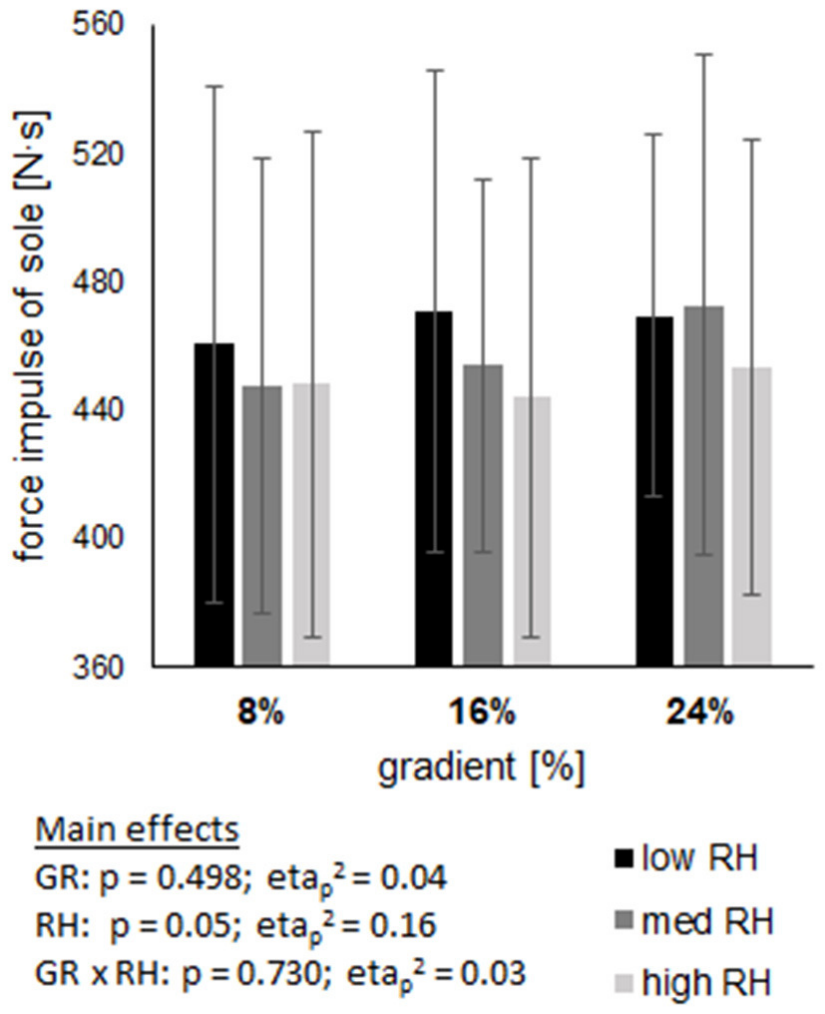
Shows the impulse of sole force across three gradients (GR), each including three riser heights (RH). Data are displayed as mean value ± standard deviation. GR × RH represents their interaction effect.

The force impulse of the foot (Figure 4) showed neither an effect of gradient (*p* = 0.498) or riser height (*p* = 0.05), nor an interaction effect of these two variables (*p* = 0.73). However, the almost significant mean change of the force impulse from low to high riser height over all gradients was −4%, revealing its relevance with a large effect (${\text{eta}}_{\text{p}}^{2}$ = 0.16). Maximum foot force was affected by gradient (*p* = 0.001) and riser height (*p* < 0.001), showing an increase from 8 to 24% gradient and a decrease from low to high riser height. Peak foot force revealed no effect for gradient (*p* = 0.292), but for riser height (*p* < 0.001), showing an increase from low to high riser height. No interaction was found for maximum and peak foot force (*p* = 0.606; *p* = 0.309).

**Figure 5:**
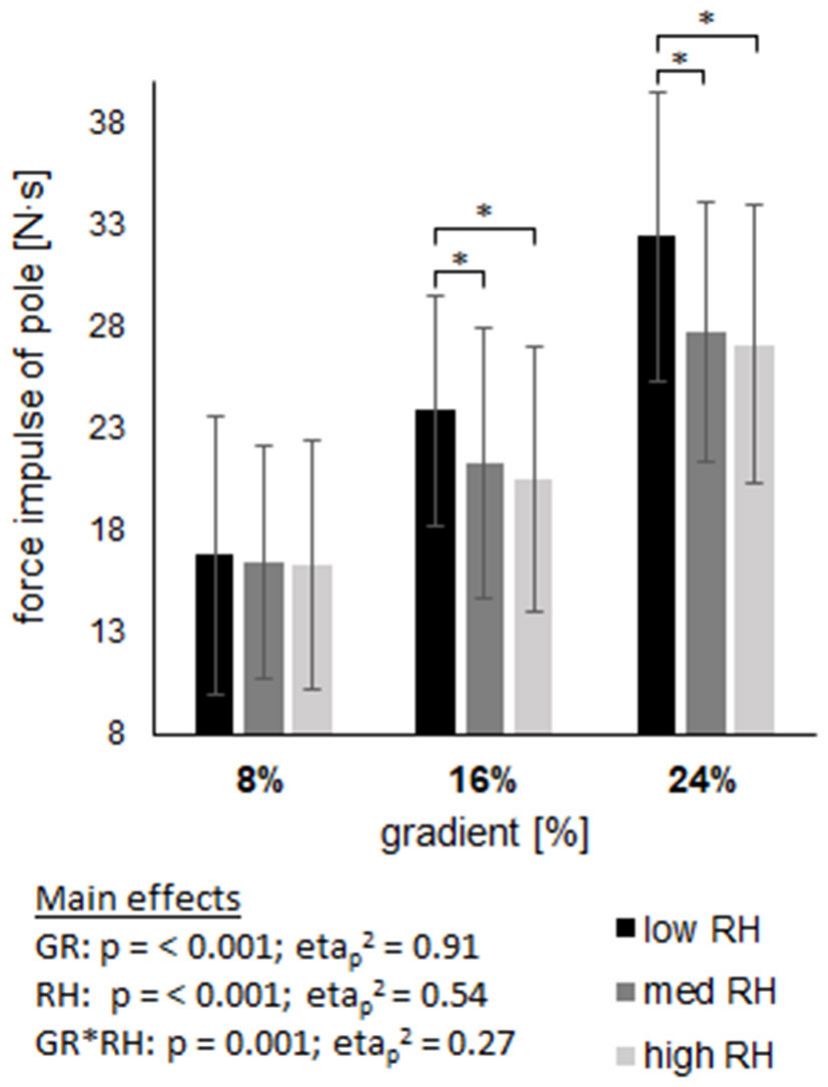
Shows the impulse of pole force across three gradients (GR), each including three riser heights (RH). Data are displayed as mean value ± standard deviation. GR × RH represents their interaction effect. The asterix (*) labels pairwise comparisons within each grade, with the level of significance (p) set at <0.05.

The force impulse of the pole (Figure 5) showed a main effect of gradient and riser height (*p* < 0.001) with an average increase from 8 to 24% gradient of 76.2%. The average decrease over all gradients from low to high riser height was −12.6%, being different within each gradient (−2.8% at 8%; −14.3% at 16%; −16.4% at 24%), resulting in an interaction effect of riser height and gradient (p = 0.001). The pole angle at initial contact was greater at 8% compared to 24% gradient (*p* < 0.001), but not affected by riser height, although a notable trend is apparent (*p* = 0.07; ${\text{eta}}_{\text{p}}^{2}$ = 0.14). The interaction of gradient and riser height (*p* = 0.036) showed an increase from low to high riser height at 8 and 24% gradient, but an unaffected situation at 16% gradient. Maximum pole force revealed a main effect of gradient (*p* < 0.001) and riser height (*p* = 0.005), but with no interaction effect (*p* = 0.07). The maximum force decreased by −4.7% from low to high riser height and increased by 59.6% from 8 to 24% gradient.

Mechanical efficiency revealed a main effect of the gradient (*p* < 0.001) increasing from 8% gradient up to 24% gradient, with no effect of riser height or an interaction effect.

### Joint kinematics

**Figure 6:**
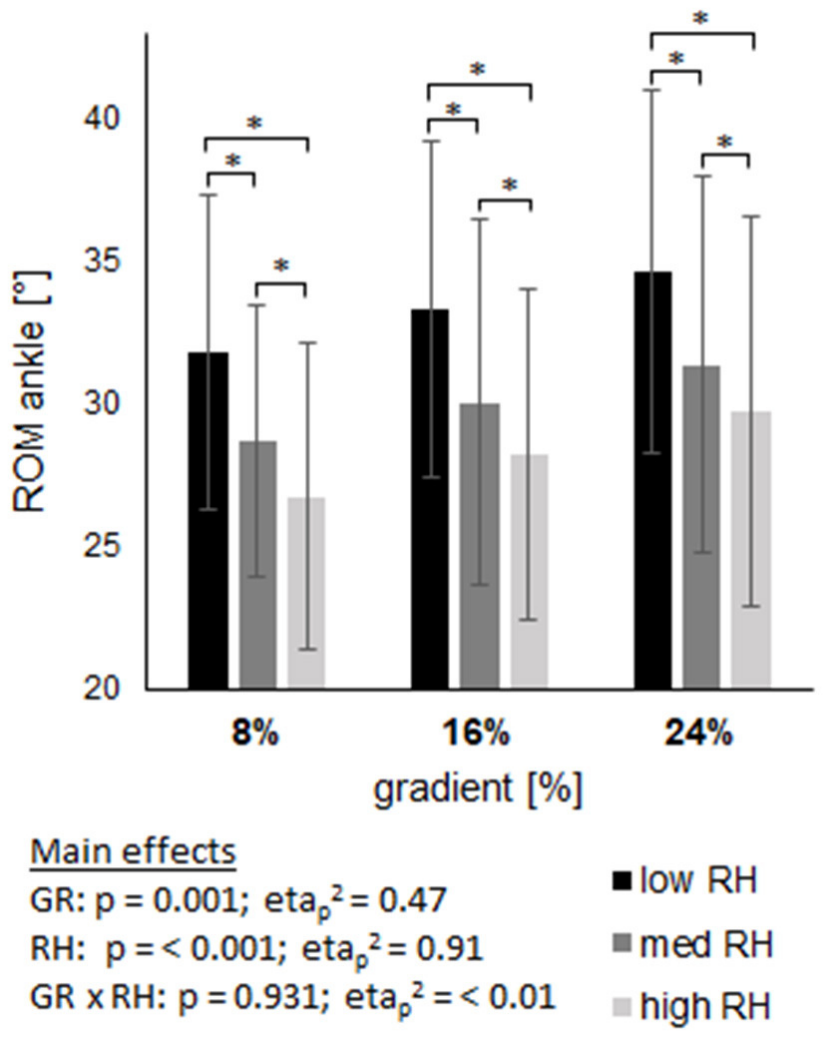
Shows the range of motion (ROM) of the ankle joint across three gradients (GR), each including three riser heights (RH). Data are displayed as mean value ± standard deviation. GR × RH represents their interaction effect. The asterix (*) labels pairwise comparisons within each grade, with the level of significance (p) set at <0.05.

**Figure 7:**
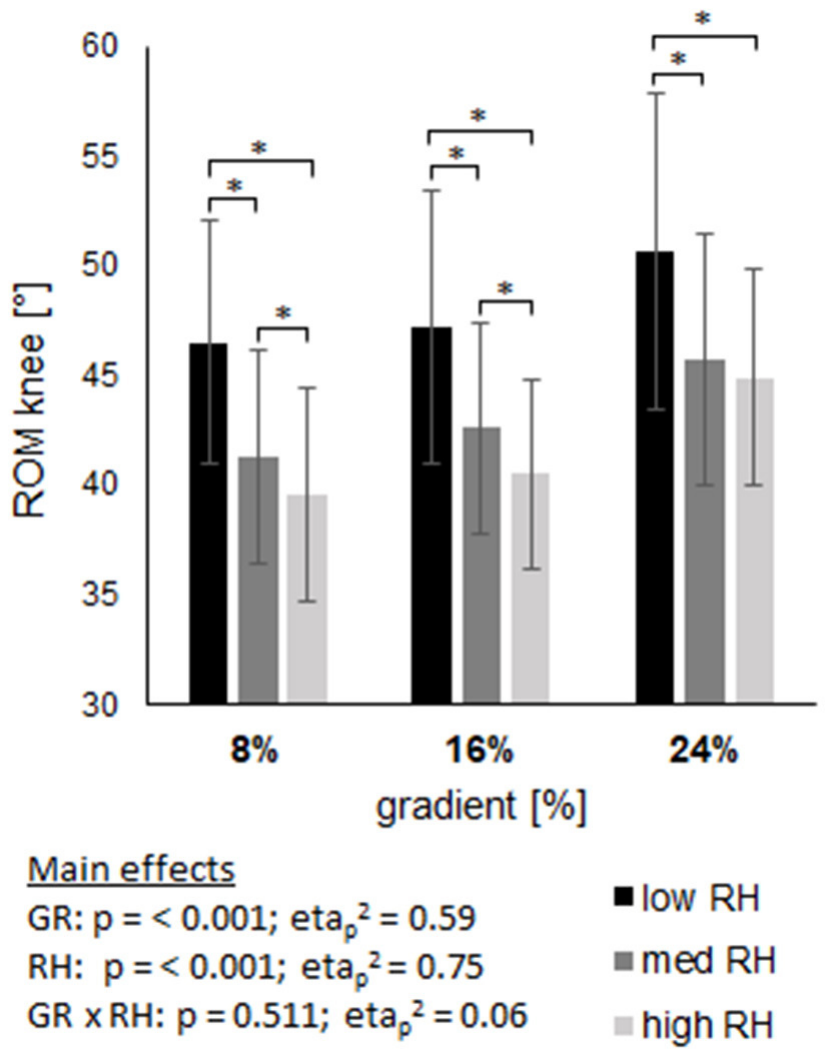
Shows the range of motion (ROM) of the knee joint across three gradients (GR), each including three riser heights (RH). Data are displayed as mean value ± standard deviation. GR × RH represents their interaction effect. The asterix (*) labels pairwise comparisons within each grade, with the level of significance (p) set at <0.05.

**Figure 8:**
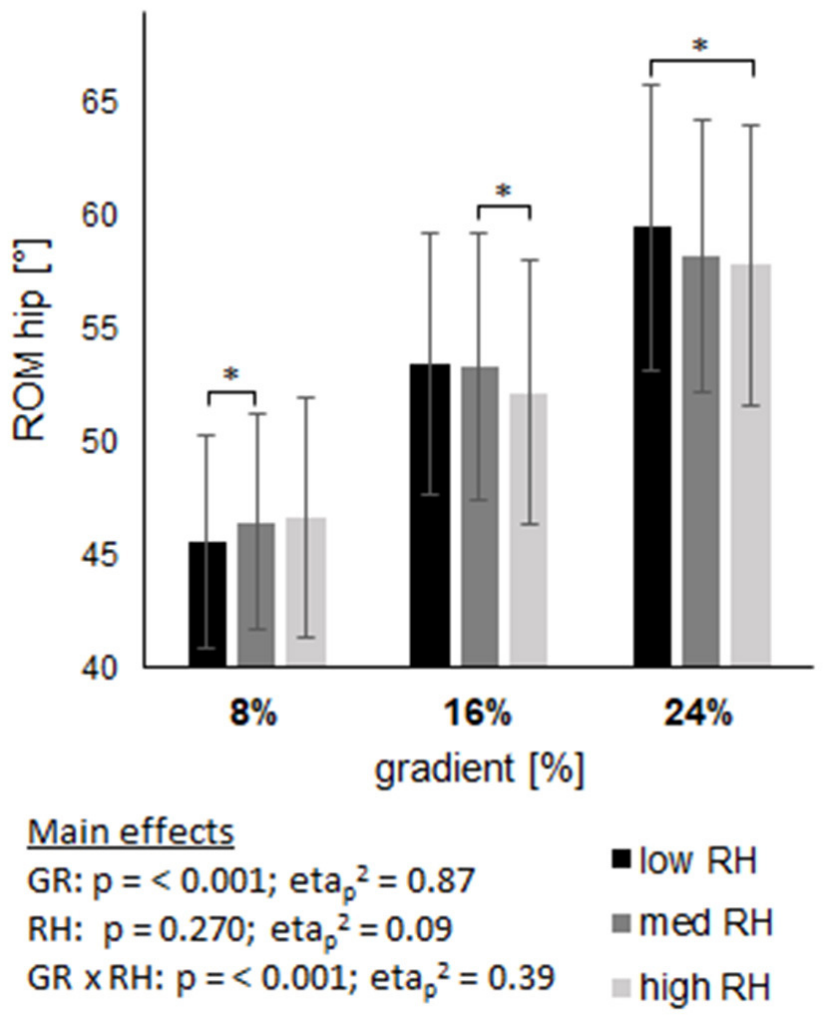
Shows the range of motion (ROM) of the hip joint across three gradients (GR), each including three riser heights (RH). Data are displayed as mean value ± standard deviation. GR × RH represents their interaction effect. The asterix (*) labels pairwise comparisons within each grade, with the level of significance (p) set at <0.05.

The ROM of joint angles are displayed in Figures 6–8, with main effects of gradient and riser height (*p* ≤ 0.001) on both, ankle and knee, while the ROM of the hip was only affected by riser height (*p* < 0.001) and not by gradient (*p* = 0.27). The high riser height reduced the ROM of ankle and knee joint compared to the low riser height, without an interaction effect (ankle *p* = 0.931; knee *p* = 0.511). The hip joint ROM revealed an interaction effect (*p* < 0.001) with an increase from low to high riser height at 8% gradient, in contrast to a decrease from low to high riser height at 16 and 24% gradient.

The foot angle is listed in Table 2, with main effects of gradient and riser height (*p* < 0.001) but no interaction effect (*p* = 0.153). Closely matching values and therefore identifiable pairs were found for (1) low riser height at 8% and medium riser height at 24%, (2) medium riser height at 8% and high riser height at 16% and (3) medium riser height at 16% and high riser height at 24%.

## Discussion

The goal of this study was to compare the influence of different riser heights and slope gradients on kinematics and kinetics during treadmill skimo. It was demonstrated that most of the parameters are influenced by both, gradient and riser height, which makes it necessary to discuss both influences and their interactions.

Gait characteristics are shown to be strongly influenced. Step frequency was increased by 5% from low up to high riser height independent of gradient and decreases by 5% from 8% gradient up to 24% gradient independent of the applied riser height. Step length data is in contrast to step frequency, since walking velocity was the same for all situations. It has also been shown in walking that the reduction of step frequency is dependent on slope gradient (Kawamura et al., 1991) and is concordant with the findings of Praz, Fasel (Praz et al., 2016a) in skimo. The change in step length is also visible in horizontal distance between the greater trochanter and the toe marker at initial contact, which describes step length in front of the body. Since the treadmill surface allows no gliding phase of the ski after initial contact, no change in step length is expected after initial contact. The fact that a higher riser height does not allow the athlete to drop the heel on the ski, restricts the ability to push the foot forward and increase ROM. This could also be discussed in relation to boot stiffness and ROM of the boot and the ankle joint, since both factors could enhance this effect. In this context we also need to differentiate between all-mountain equipment and racing equipment. While racing equipment is built to be as light as possible with little friction and resistance at the boots' pivot point at the ankle, all-mountain equipment targets other main objectives like thermo-insulation or comfort and has more resistance when rotating the boot's cuff. It might be inevitable to adopt the riser height with a stiff boot to compensate for the lack of boot flexibility.

The analysis of step length agrees with gait analysis, where the high riser height was shown to provide a shorter swing time and shorter single stance phase, reasoned by an earlier heel contact with the binding at foot strike. Additionally, ROM of the ankle and the knee were directly affected by the change in riser height. Figures 6, 7 show the reduction of the joints' ROM when using a higher riser height, independent of gradients. These interpretations are also supported by the ROM of the hip (see Figure 8) at 16 and 24% gradient, where a significantly lower ROM when using the high riser height was found. The hip is not extended maximally because the heel is elevated during push off, which lowers the ROM of the hip and consequently step length as well. We interpret the evident interaction and inverse shape of hip ROM change at 8% gradient by a counterproductive limitation in ROM of the knee and ankle joint due to the high heel, which then is compensated by the hip. In general the boot is reported to have a ROM of 74°, while we found an average ankle ROM from 27 to 35° at various situations, which excludes the boot being a limiting factor concerning ankle movement.

These results, supported by the highest effects of riser height setting within gradients for step characteristics, trochanter to toe distance and gait analysis found at 8% gradient, lead to the conclusion that the movement pattern is influenced the most by the different riser heights at the lowest tested gradient. Additionally, the comfort scale clearly demonstrates the highest riser height being the most uncomfortable choice at 8% gradient.

Pole force application may also be more effective at the lower riser height. On one hand, impulse of pole forces and maximum pole forces were higher when using the low riser height compared to the high riser height. On the other hand, pole angles at initial contact of the pole were lower when using the low riser height, at 8 and 24% gradient. This combination of a more advantageous direction of force application, higher force values and higher force impulse indicate stronger propulsion. Nevertheless, a flatter pole angle is not only affected by riser height, but also by gradient. This reveals a more beneficial value in terms of propulsion at 24% compared to 8% gradient. The flatter pole angle at initial contact can be linked to a flatter torso angle occurring at steeper gradients. With these benefits occurring at 24%, it is an advantage to choose this gradient compared to lower gradients. This finding is in concordance with the vertical energy cost analysis of Praz et al. (2016a,b), who also suggested to choose a steeper gradient if possible.

In the analysis of foot pressure, it was necessary to analyze peak and maximal pressures separately. Peak force describes first peak in the gait cycle, as a result of initial contact, which is also known as braking force during running (Heiderscheit et al., 2011). Maximum pressure occurred typically during push off phase. Both events, initial contact and push off, were demonstrated to be influenced by riser height. Even though single values do not represent the course of the occurring force, they indicate a probable shift or a trend. Although we cannot address the overall braking or propulsive forces, peak values indicate higher peak braking force for the high riser height compared to the low riser height. This could be explained by the fact that there was earlier heel contact when using the high riser height, which resulted in a higher force value. However, based on that, the direction of force was not measurable with the current setup, it is possible that the peak value, due to the forward inclined foot when using the high riser height, also creates a propulsive force. This would be comparable to the potential effect of heel to toe drop in running shoes (Richert et al., 2019; Mo et al., 2020).

It was demonstrated that foot angle is well comparable for certain combinations of gradient and riser height (8% low vs. 24% medium, or 8% medium vs. 16% high, or 16% medium vs. 24% high). While for example ankle ROM or cycle timing parameters show similar results for the mentioned pairs of a quasi-similar position of the foot in space, the analyzed force parameters do not necessarily confirm this similarity. Since the walking speed was the same for all situations, a higher strain existed for higher gradients and therefore similar kinematic parameters resulted in different kinetic measurements. Comfort scale could demonstrate the supremacy of the low riser height at 8% gradient and the medium riser height at 16 and 24% gradient, whereby a foot angle between −5° and 0° was proven to be the most comfortable choice.

As a link between biomechanical measurements and physiological responses, mechanical efficiency was calculated. Even though various biomechanical parameters showed an effect of the heel riser height, the mechanical efficiency was not affected. Nevertheless, mechanical efficiency was affected by gradient. Similar to Praz et al. (2016a,b) we found mechanical efficiency being higher at steeper gradients.

### Limitations

Even though we used standard skimo equipment, walking on the treadmill is somewhat different compared with walking on snow. However, a direct comparison of walking on snow and on the treadmill would be necessary to allow the transfer of results directly to the field. Additionally, the treadmill was limited in maximum gradient (24%) which can be judged as a medium gradient when skiing outdoors. Therefore, analysis of steeper slopes is warranted. Our protocol used the same speed for all gradients, which is an advantage when comparing biomechanical parameters, since the same walking speed was compared throughout the protocol, however, the strain on the participants was different across the three slope gradients. In particular, this difference in exercise intensity at different gradients could influence the locomotor patterns.

## Conclusion

The purpose of this study was to compare the influence of riser heights and slope gradient on biomechanical variables during treadmill skimo and to learn more about the human-equipment interaction in skimo. Adjusting the riser height, dependent of the slope gradient, influences human–equipment interaction and changes movement patterns. Independently of the gradient, step frequency was increased by 5% when comparing the high riser height with the low riser height. In contrast to the low riser height, the high riser height increases the heel to toe drop during stance phase, which shortens step length, reduces swing time and reduces ROM of the knee and ankle joint. Additionally, the poles' impulse of forces and the pole angle are more beneficial when using the low riser height at the analyzed gradients. Mechanical efficiency suggests steeper gradients being more beneficial, while no difference concerning the applied heel riser heights was found. However, this does not exclude physiology being affected in any other way.

Since the high riser height indicates to influence the movement pattern negatively, especially at low gradients, we suggest applying the low riser height when ascending a gradient of 8%, while existing effects and interaction effects suggest changing toward the medium riser height when ascending the steeper tested gradients. This recommendation is supported by the comfort scale, which indicates the low riser height being most comfortable at 8% gradient and the medium riser height being most comfortable at 16% and 24% gradient. We did not find an indication for the highest riser height being the best choice at the analyzed gradients—not even the steepest tested gradient.

## Data availability statement

The raw data supporting the conclusions of this article will be made available by the authors, without undue reservation.

## Ethics statement

The studies involving human participants were reviewed and approved by Ethical Committee of the University of Salzburg Universität Salzburg Kapitelgasse 4-6 5020 Salzburg, Austria. The patients/participants provided their written informed consent to participate in this study.

## Author contributions

ML, JS, TS, and A-MW contributed in an equal way to the conceptual and planning phase of the study. ML, JS, and A-MW were involved in data collection and annotated and improved this paper. ML and A-MW analyzed the presented data. ML wrote the first draft. All authors contributed to the article and approved the submitted version.

## Conflict of interest

Author TS is employed by Red Bull Athlete Performance Center, Salzburg, Austria. The remaining authors declare that the research was conducted in the absence of any commercial or financial relationships that could be construed as a potential conflict of interest.

## Publisher's note

All claims expressed in this article are solely those of the authors and do not necessarily represent those of their affiliated organizations, or those of the publisher, the editors and the reviewers. Any product that may be evaluated in this article, or claim that may be made by its manufacturer, is not guaranteed or endorsed by the publisher.
